# Diatom microalgae as smart nanocontainers for biosensing wastewater pollutants: recent trends and innovations

**DOI:** 10.1080/21655979.2021.1996748

**Published:** 2021-12-07

**Authors:** Mohd Jahir Khan, Anshuman Rai, Ankesh Ahirwar, Vandana Sirotiya, Megha Mourya, Sudhanshu Mishra, Benoit Schoefs, Justine Marchand, Shashi Kant Bhatia, Sunita Varjani, Vandana Vinayak

**Affiliations:** a Diatom Nanoengineering and Metabolism Laboratory (DNM), School of Applied Science, Dr. Harisingh Gour Central University, Sagar, India; b School of Engineering, Department of Biotechnology, Mmu, Deemed University, Ambala,India; c Metabolism, Bioengineering of Microalgal Metabolism and Applications (MIMMA), Mer Molecules Santé, Le Mans University, Le Mans, France; d Department of Biological Engineering, Konkuk University, Seoul, Korea; e Paryavaran Bhavan, Gujarat Pollution Control Board, Gandhinagar, India

**Keywords:** Biosensors, Diatoms, Microalgae, Nanomaterials, Pollutants, Wastewater

## Abstract

Microalgae have been recognized as one of the most efficient microorganisms to remediate industrial effluents. Among microalgae diatoms are silica shelled unicellular eukaryotes, found in all types of water bodies and flourish very well even in wastewater. They have their silica cell wall made up of nano arrayed pores arranged in a uniform fashion. Therefore, they act as smart nanocontainers to adsorb various trace metals, dyes, polymers, and drugs which are hazardous to human as well to aquatic life. The beautiful nanoarchitecture in diatoms allows them to easily bind to ligands of choice to form a nanocomposite structure with the pollutants which can be a chemical or biological component. Such naturally available diatom nanomaterials are economical and highly sensitive compared to manmade artificial silica nanomaterials to help in facile removal of the toxic pollutants from wastewater. This review is thus focused on employing diatoms to remediate various pollutants such as heavy metals, dyes, hydrocarbons detected in the wastewater. It also includes different microalgae as biosensors for determination of pollutants in effluents and the perspectives for nanotechnological applications in the field of remediating pollutants through microalgae. The review also discusses in length the hurdles and perspectives of employing microalgae in wastewater remediation.

## 1. Introduction

Recent years, application of microalgae in the treatment of wastewater has increased remarkably. This is due to the fact that wastewater contain nutrients required for the growth of microalgae [1,2]. These nutrients mainly include nitrates (N), phosphates (P), and trace elements, essential for the growth of microalgae [3]. Since most of the microalgae are photosynthetic in nature, they do not need external organic compound for their growth and replication, whereas some strains could avail organic compounds in the absence of light too. Therefore, microalgal bioremediation could be combined with traditional and single biological treatment methods for efficiently treating wastewater [4,5]. Among microalgae both *Chlorella* and *Micractinium* showed good response in removing nitrates and phosphates from wastewater [6,7]. They were cultivated in wastewater in order to check the kinetics of nutrient removed and their growth [8]. About 95% of the soluble phosphorus (P) was removed, and soluble nitrogen (N) was reduced up to 95.7% and 93.9% for *Chlorella* and *Micractinium*, respectively. Soluble nitrogen decreased by 50.6% for *Chlorella* and 45.7% for *Micractinium* in primary effluent mixtures. Higher concentration of N and unbalanced N/P ratios in the mixed wastewater resulted in higher removal rates of N and potassium (K) accompanied by rapid uptake, simultaneously resulting into algae growing in the environment rich in extracellular proteins [8]. In a study of four different types of wastewater samples *viz*; before primary settling (type 1); after primary settling (type 2); wastewater after activated sludge tank (type 3); and centrate (type 4) were tested to observe the growth of green algae *Chlorella sp*. It was done to check how the *Chlorella sp*. removed N, P, chemical oxygen demand (COD), and trace metals from these four different samples of wastewater [9].

In case of wastewater after activated sludge tank (type 3), the major inorganic nitrogen (NO_3_–N) formed was 62.5% of which 6.3-fold NO_2_-N was removed. The average specific growth rates in the different wastewater type 1, 2, 3, and 4 at exponential period was 0.412, 0.429, 0.343, and 0.948, day^−1^ respectively. Algal growth increased in the centrate (type 4) because of higher levels of N, P, and COD compared to that in other three wastewater types, while the treatment of wastewater types 1, *i.e*. before and type 2, *i.e*. after primary settling was more systematic in nutrient depletion than that of pollutants. Metal ions like Fe, Ca, Mg, Mn, and Al in type 4, *i.e*. centrate were removed efficiently by algae growing in it [9]. In addition, it has been seen that among bacteria genetically engineered cells of *Deinococcus radiodurans* nonspecific for nitrogen phosphatase removes 70% of uranium from 1 g uranium/g dry weight cells [10]. Table 1 shows removal of different heavy metals by different microalgae at different efficiencies which vary obviously on the type of wastewater and type of microalgae strain.

**Table 1.** Microalgae for metal removal from wastewater

Metals	Microalgal strain	Removal efficiency (%)	References
Copper, zinc, cadmium, and mercury	*Cladophora fracta*; live algae	Cu^2+^, Zn^2+^, Cd^2+^, and Hg^2+^ were 99%, 85%, 97%, and 98%, respectively	[11]
Cadmium	*Scenedesmus sp*. *Chlorella sp*. *Chlorella vulgaris*	73% 33–41% 66%	[12]
Chromium	*Chlorella vulgaris* *Scenedesmus sp*. *Spirulina sp.*	50.7–80.3% 92.89% 82.67%	[13–15]
Copper	*Spirulina maxima* *Chlorella vulgaris* *Scenedesmus obliquus*	94.9% 96.3% 72.4–91.7%	[16,30]
Lead	*Chlorella vulgaris* *Chlorella sp*. *Pseudochlorococcum typicum*	89.26% 66.3% 70%	[17,18,32]
Mercury	*Chlorella vulgaris* *Chlorella vulgaris* *Pseudochlorococcum typicum*	79–86% 34.21–93% 97%	[18–20]
Nickel	*Scenedesmus sp*. *Chlorella vulgaris* *Chlorella miniate*	97% 33–41% 60-73%	[12,21]
Zinc	*Chlorella sp* *Synechocystis sp* *Scenedesmus sp*	60–70% 40% 98%	[21,32]
Cadmium, Copper, Lead, and Zinc	*Haslea ostrearia, Phaeodactylum tricornutum, Skeletonema costatum, and Tetraselmis suecica*; live algae	Reduction in Cu and Cd	[22]
Chromium	*Chlorella miniata; dried algae; cultivated in domestic wastewater*	75% and 100% removal for Cr (III) and Cr (VI), respectively	[23]
Lead, Cadmium, Copper, and Arsenic	*Cyanophyta (Oscillatoria princeps 92%, Oscillatoria subbrevis 2%, and Oscillatoria formosa 1%) and Chlorophyta (Spirogyra aequinoctialis 3%, Mougeta sp. 1%, and others 1%);* dried algae	Metals were removed	[24]

In yet similar study microalgae *Chlorella* was grown in only bold basal medium (BBM), wastewater, and wastewater enriched with BBM elements [25]. It was observed that wastewater enriched with BBM elements showed highest growth in *Chlorella* cell density; however, due to rapid growth there was decrease in lipid accumulation. On the offset, wastewater showed maximum lipid accumulation of about 16.2 mg L^−1^ day^−1^, with removal of NO_3_-N, NH_3_-N, and TP, to an extent of 74.2, 83.3, and 78.0%, respectively. However, BBM media showed additional recovery of monounsaturated fatty acids without any nutrient recovery. Hence proving that microalgae grown in wastewater were very economical way of bio cycling important nutrients from wastewater while simultaneously producing lipids and biofuel [25,26]. On the other hand, phycoremediation of tannery wastewater (TWW) with *Scenedesmus sp* showed significant results. In this study, *Scenedesmus sp*. was grown in TWW under controlled laboratory environment which showed significant results on the 12^th^ day of harvesting. The results showed that, during growth period the accumulated algal biomass decreased percentage amounts of heavy metals (Cu-73.2–98%, Cr-81.2–96%, Zn-65-98%, and Pb-75-98%) and nutrients (PO_4_ > 95% and NO_3_ > 44.3%). Thus showing that *Scenedesmus sp*. has potential for biomass production and also ability to phycoremediate the toxic pollutants found in TWW [27,28]. Another microalgae *Coelastrum sp*. has great future to produce large amounts of biomass while simultaneously removing nutrients from wastewater [29]. In this study outcome of different Soluble Chemical Oxygen Demand (sCOD) concentrations and light intensity was done on the total Kjeldahl nitrogen (TKN), nitrate and total phosphorus (TP) removal, microalgae growth, and CO_2_ utilization rate. It was observed that maximum growth of about 2.71 g L^−1^ day^-1^and CO_2_ removal of 53.12 mg L^−1^ day^−1^, took place at light intensity of 6900 Lux with wastewater having initial sCOD of 750 mg L^−1^. However, on 4^th^ day *Coelastrum* sp. showed the removal efficiency of sCOD 53.45%, 91.18% TKN, 87.51% nitrate and 100% of TP. In addition, when *Coelastrum* sp. was cultivated under semi-batch conditions with continuous gas flow, the biomass productivity was seen to be 0.281 g L^−1^ day^−1^. In addition, under the same condition, the average removal rate of TKN, TP, N, and sCOD was 83.51%, 100%, 80.91%, and 41.4%, respectively, by this microalgae. These results suggest that *Coelastrum* sp. is a befitting microalgae for carbon dioxide bio fixation, and wastewater bioremediation [29]. Furthermore, a study on treating toxicity of metals like Cu is done by sulfate reducing bacteria (SRB) *eg. Desulfovibrio sp*. using novel sulfur reduction bacteria (SRB) microalgal spheres employing four microalgae *viz; Scenedesmus obliquus, Selenastrum capricornutum, Chlorella vulgaris* and *Anabaena spiroides* [30]. These microalgae were used as source of carbon to treat Cu (II) effluent wastewater at a concentration of 60 mg L^−1^ and 600 mg L^−1^ sulfate. The microalgae are broken down into fatty acids by sulfate reducing bacteria, which serve as carbon source for sulfur reduction bacteria (SRB) so as to lower the COD. Furthermore, it has been observed that immobilized SRB nutrient beads showed efficient remediation than free cells, because the beads not only secured SRB against toxicity by heavy metals but also increased the COD/SO_4_^2−^ ratios. Results showed that after 45 days, the Cu (II) elimination rates increased from 91.7 to 98.2% and sulfate removal rates increased from 72.4 to 74.4%. The outstanding efficiency and strength of this immobilized SRB microalgae bead provides an encouraging strategy to deal with pollution caused by heavy metals instead of using free microalgal cells [30]. *Chlorella sp*. regulates high removal of Zn^2+^ and has shown its 60–70% removal from culture medium containing from 5 to 20 mgL^−1^ Zn^2+^ hence reducing it to 42% at 50 mgL^−1^. However, a continuous fall in cell number from 538 × 10^5^ to 8 × 10^5^ cells mL^–1^ suggested Zn toxicity in *Chlorella*. The growth retardation in microalgae cells is dependent not only upon the amount of heavy metal ions immobilized on its surfaces but also on its amount present intracellularly [31] while in case of Zn, retardation in growth may be due to Zn ions present extracellularly. In the algal stabilization ponds, Zn^2+^ and Pb^2+^ were removed by 72% and 73%, respectively. These results display a good treatment capacity of algae stabilization pond system by *Chlorella* sp. along with remediation of toxic heavy metals [32]. It has been observed that the cosmopolitan microalgae *Chlorella sp*. assembles and remediates toxic heavy metals from the wastewater [33]. This is because heavy metals such as Cu, Zn, Fe, Co, Mo, and Mn are required as essential nutrients for its growth and multiplication. *Chlorella* shows adsorption of heavy metals found as pollutants in tannery industries [34]. The reduction of different metal ions was Pb 12.54 mg L^−1^; Co 7.37 mg L^−1^; Cr 10.92 mg L^−1^; Ni 9.15 mg L^−1^; Cd 8.48 mg L^−1^; Cu 10.71 mg L^−1^ Zn 11.56 mg L^−1^ at 20^th^ day of the treatment [35]. The maximum bioadsorption capacity of *Chlorella* to remove heavy metals was found to be 83.43, 82.15, 63.29, 58.92, 70.53, 64.83, and 81.36, µg L^−1^ for Zn, Ni, Cd, Co, Pb, Cu, and Cr respectively. This was followed by optimal yield of value added products as well as cleaning of wastewater effectively yet economically especially if microalgal value added has high industrial demand and gives high yield in the presence of high light and other components which are freely available. For instance astaxanthin in green-red microalgae *Haematococcus pluvialis* and fucoxanthin in diatoms [36,37]. On the other hand, various studies showed that than other microalgae are also used widely in wastewater treatment such as *Chlorella, Arthrospira* [38], *Scenedesmus* [39], *Botryococcus* [40], *Chlamydomonas* [41], and *Phormidium* [42]. Brown microalgae has great potential in treating wastewater alone or in combination with bacteria [43]. Among the commonly existing microalgae used for wastewater treatment, optimal yield of value added products as well as cleaning of wastewater effectively yet economically is very important. Furthermore, if microalgae has high value added for instance astaxanthin in green-red microalgae *Haematococcus pluvialis* and fucoxanthin in diatoms. Among these two microalgae diatoms are robust microalgae due to their silica structure which not only remediate wastewater rich in micronutrients and macronutrients required for its growth but also acts as smart nanocontainers. Consequently, oxygen produced by diatoms during photosynthesis encourages heterotrophic bacterial growth which leads to enhanced degradation of organic matter including heavy metals by bacteria. They thus play an important role in biosensing pollutants in untreated sewage sites rich in pharmaceutical drugs, NH_4_^+^ and PO_4_^3-^ [44]. On the offset diatom bio-monitor wastewater rich in heavy metals like Cr, Pb, Cd, Cu by not only showing morphological changes in their silica frustule but also show significant physiological changes. They also effectively bio remediate not only heavy metals but also wastewater from different sources like dairy waste [45], dyes waste [46–48], and piggery waste [49] while simultaneously yielding value added products [49] and recycling the wastewater. In this article light has been thrown on the ability of diatom to bioremediate wastewater and biosense pollutants in it owing to its magnificent silica nanostructure which act as a nanocontainer for wastewater pollutants. The use of diatoms as nanomaterials alone or in conjunction with conjugate linkers has been elaborated. The teratological deformation of diatoms morphological outlines due to heavy metals has shown lipid stress and enhancement in lipid bodies has been explained. Besides this the diatoms as microfluidic devices to biosense the wastewater pollutants based on biosensor technology along with the hurdles and future research has been discussed in length.

## 2. Diatoms silica shells as robust nanomaterials to treat heavy metals in waste water

Diatoms are golden brown algae, responsible for fixing about 25% of atmospheric CO_2_ [50]. They exist in different shapes and sizes ranging from 2 to 200 µm which are identified both morphologically and at molecular level [51]. There are about 20,000 species of diatoms with pores arranged in a beautiful and rigid walled nanoarchitecture [52–54]. These pores range in size from 3 to 50 nm in diameter [55]. Besides being found in all types of water bodies they are photosynthetic, convert atmospheric CO_2_ to carbohydrates, thus forming different biomolecules like proteins, lipids, carotenoids, *etc.* [56]. They are high source of naturally occurring value added products besides having immense applications in nanotechnology and sensing water quality of organic pollutants, metals, dyes, pesticides, hydrocarbons, and drugs *etc*. in aquatic ecosystems [50,57,58]. They are thus widely studied for their role as indicators of different kinds of water pollution, and their phytoremediation in the initial phase [59]. Since rapid industrialization and urbanization are the main reason for groundwater contamination andwater deficit [60,61]. There are various technologies to treat wastewater, however, each has its stumbling block and asset [62]. Therefore, it’s important to choose the one that could purify the wastewater and recover the resources from it. Since the disposal of wastewater into the water bodies results in the release of nutrients, this causes eutrophication in water bodies [63]. Furtheromre, heavy metals like Cu, Cd, Tl, Hg, As, Zn, Ni, Cr, and Pb in wastewater are harmful pollutants even in traces. The destructive nature of heavy metals is because of their bio toxicity in biotic systems [64]. Not to limit heavy metals, even dyes are the most common pollutants present in wastewater which is very important to remove or treat for clean environmental [65]. Therefore, it is is essential to not only utilize the nutrients present in wastewater but also remediate the heavy metals in it.

### 2.1 Diatoms as nanoabsorbents to remediate heavy metals

Recent study showed that the nanomaterials acts as sorbents for removing toxic heavy metals from wastewater due to their high surface to volume ratio, porous nature, and reactivity thus acting as suitable nanoadsorbents [66]. The selectivity and adsorption capacity of nanomaterial effectively eliminate heavy metals from water, even in trace concentrations [67]. The use of natural adsorbents to remove hazardous metals is both cost effective and environmentally beneficial. The treated clay minerals remove more metal ions under the same circumstances as untreated clay minerals due to increased surface area [68]. The immobilization of cement treated clay at different concentrations was tested and found that concentration of 6% and 9% was efficient in removing Pb^+2^ metal ions. The removal of Pb^+2^ increased with the increase in amount of cement in the clay. Furthermore, for the removal of heavy metals from wastewater, several remediation methods have been developed, the most common of which are filtration, oxidation and reduction, sedimentation, media filtration, and membrane filtration. Furthermore, heavy metals are unable to dissolve in flowing water and overcome variations in pH, conductivity, or microbiology that impact water quality. However, most of the techniques applied for removing heavy metals from wastewater are costly, thus adding financial burden [69]. Furthermore, owing to the specific properties of mesoporous silica materials they can be used as propitious adsorbents and catalytic support for treating wastewater [70,71]. Since manufacturing silica is arduous and costly and include toxic materials, there is a high demand to substitute these synthetic materials with steady and natural surrogates. On the offset, nature has treasured three-dimensional (3-D) porous silica structures known as diatoms, the single-celled photosynthetic microalgae [72] which are the rich source of natural porous silica [73].

The diatoms nano shells in conjugation with the ligands can be seen to adsorb toxic metals more in salinity-dependent marine diatoms as the cells at low salinity develop stronger affinity for adsorption of Cd^++^ [74]. It is observed that diatom cells thriving at lower salinity had a lower surface potential, higher specific surface area, and more sulfur-containing groups in the cell wall, followed by stronger Cd binding capacity in the cells. Hence, when the salinity decreases, more Si is found as poly-silicic acid. Thus change of Si content and speciation in the cell wall are considered as a key factor for variations of adsorption of Cd on its surface [75].

### 2.2 Teratological changes in living diatoms found in wastewater

Diatoms have capability to phycoremediate different kinds of wastewater due to their implicit cellular mechanisms. Furthermore, diatoms aid to rejuvenate the past climatic conditions by indicating water quality of water bodies. Silicified diatomaceous cell walls deform rapidly and characteristically in presence of chemical contamination, such as alterations in cell size, outline of frustules, patterns of raphe, and stria [76,77].

On the offset, diatoms flourish well in wastewaters by utilizing several macronutrients such as carbon, nitrogen, phosphorus, silica, and micronutrients such as iron, magnesium, calcium, potassium, manganese, copper, and cobalt required for their growth and photosynthesis to form biomass [78,79]. They not only utilize metals as nutrients but also remediate dyes, drugs, *etc*. Furthermore, exopolysaccharides secreted by microalgae prevents and slows down the leaching and chemical breakdown of plastics into micro and nanoplastics thus serving as biofoulant [80–82].

**Figure 1.**
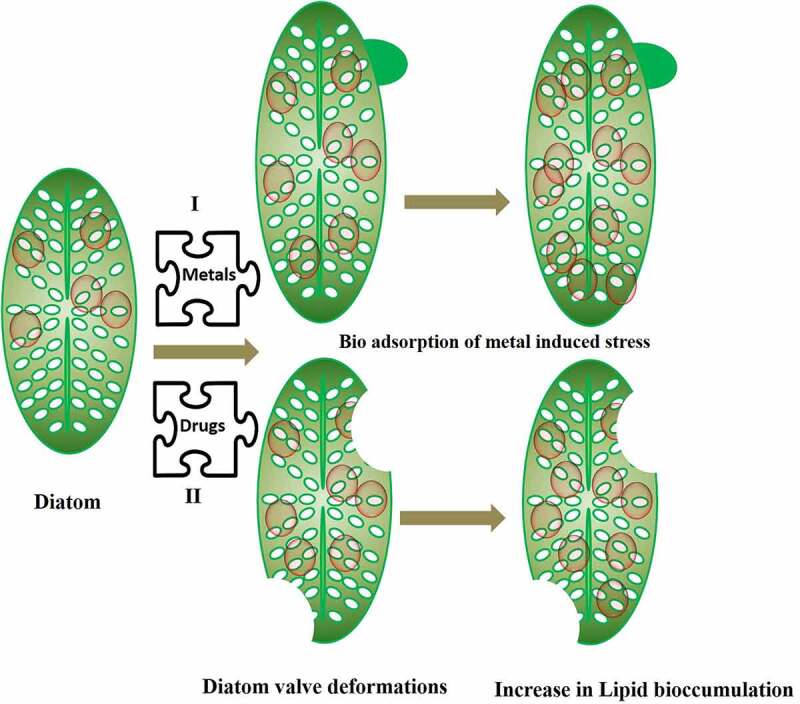
Diatoms undergoing valve deformation and increase lipid accumulation under stress conditions during remediation of pollutants

Additionally, the diatoms have a large impact on the biogeochemical cycles of carbon, phosphate, nitrate, and silica [83,84]. As a result, this mineral’s absorption is critical. The species-specific patterning of the silicon cell wall is the most distinguishing characteristic of a diatom [85]. When subjected to various types of stress throughout the reproductive process, the diatom cell outline and their frustule pattern can alter in a variety of ways, resulting in teratological forms. These changes might be minor, making it difficult to distinguish between normal and teratological cells. They can be so pronounced that it is impossible to determine if an unfamiliar form is teratological or belongs to a new species or variation. Teratological forms emerge as an unintended consequence of environmental stressors, which can be both physical and chemical in nature. Morphological variations in diatoms are often adaptive or nonadaptive in response to different environmental conditions [76]. Teratological forms are nonadaptive phenotypical abnormalities that typically have an effect on the contour of the valves or their stripe pattern. The rise in temperature can also affect the volume of the diatoms and reduce the size of the cells, although it is not the only relevant parameter [86]. This characteristic has no clear and universal influence on the carbon and nitrogen uptake of larger diatoms [87]. On the other hand, the conductivity influences the diatoms to a considerable extent. Diatoms can be attacked by metal contamination such as Ge, Cu, and Zn, which intervene silicate pathway in the diatoms [88]. For example, it was seen that *Gomphonema pseudoagur* found in Saraswati River Kurukshetra showed teratological deformation due to heavy metals [89]. The inductively coupled plasma mass spectrometry (ICP-MS) analysis of the site showed contamination by heavy metals, mainly Pb and Se. The deformity was further associated with high lipid content. Hence, the heavy metal pollutants result into valve or teratological deformation in diatom frustule. The morphological deformations not only make identification difficult but puts the diatom under metabolic stress by increasing the lipid bioaccumulation. Table 2 and Figure 1 shows how different heavy metals which are pollutants found in wastewater alter the morphology of diatoms by bringing undulating changes in frustule, stria, raphe and valve in diatoms and increasing the bioaccumulation of lipids.

**Table 2.** Deformed diatoms on exposure to metals

Diatoms	Teratology	Pollutant	References
*Nitzschia fonticola*	Deformed raphe channel	Zinc and copper	[90]
*Achnanthidium pyrenaicum*	Abnormal outline	Fluoranthene (pesticides)	[91]
*Amphora pediculus*	Distorted outline	Cadmium	[92]
*Fragilaria capitellata*	Deformed valve outline	Pesticides	[93]
*Nitzschia amphibia*	Distorted outline	Cadmium, arsenic, lead, mercury, copper, zinc	[94]
*Fragilaria nanoides*	Deformed central area	Copper	[95]
*Eunotia sp.*	Distorted outline (twist in middle of the valve)	Copper	[95]
*Fragilaria recapitellata*	Distorted valve outline	Zinc and copper	[96]
*Reimeria uniseriata*	Deformed valve outline	Cadmium, arsenic, lead, mercury, copper, zinc	[90]
*Discostella pseudostelligera*	Deformed striation pattern in the side of the valve	Cyanide and heavy metals	[97]
*Eunotia spp.*	Bent or dilate, absence of symmetry	Copper, iron, zinc, nickel	[98]
*Brachysira microcephala*	Distorted outline	Cadmium, zinc, lead	[77]
*Phaeodactylum tricornutum*	Abnormal boundaries or wrinkled shaped	Copper and zinc	[99]
*Gomphonema pseudoaugur*	Dilated in the middle of the valve	Lead and selenium	[89]

### 2.3 Effect on lipid bodies

Diatom showed large amount of lipid body (LB) accumulation under metal stress compared to the diatom living under standard conditions. It had been observed that fatty acid and sterol content reducedin the marine diatom *Asterionella glacialis* under Hg and Cd stress [77]. Although, the specific process of LB production in diatoms is unknown, research has shown that metal stress disrupts the diatom cell’s cytoplasmic homeostasis, which may enhance their sinking rate. Even though it’s not the case with all diatoms, as diatom *Diadesmis confervacea* has been discovered with oil oozing property in a clean fresh water lake of Haryana, India, without any heavy metal contamination [100]. Thus, increasing lipid content inside diatoms *via* LB production enhances buoyancy and decreases diatom sinking [101]. In a study by [102], it was shown that parameters like motility, LB, size reduction, and deformation of diatoms serves as biological monitoring tool for heavy metal detection [102]. In a study, it has been shown that there is a correlation of change in diatom morphology and lipid bodies (LB) as a function of metal concentration in the Khetri and Zawar regions in Rajasthan, India containing Cu and Zn [103]. All the heavily metal polluted sites showed increase in LB in about 8 diatom species *viz; Craticula cuspidata, F. capucina, Navicula subminusula, Nitzschia amphibia, Nitzschia gracilis, Nitzschia linearis, Nitzschia sigmoidea,* and *P. conica* found in these regions. As per available literature LB in diatoms increases under nutrient stress and its accumulation in metal stress is not clear. On the offset, deformities in about 20 diatom species were observed in Khetri and Zawar regions rich in Cu and Zn. The deformation in diatom raphe was seen in Cu polluted sites compared to Zn rich sites. Further these sites showed absence of centric diatoms and its species for, e.g. *Acanthedium minnutissium* showed its abundance in Zawar region than at Khetri. This is well proved by the fact that *A. minutissimum* is tolerant to metals like Cu, Fe, Pb, and Zn. Apart to this, reduction in diatom size was observed which was independent of the type of diatom and its morphology.

Pearson’s correlation analysis showed that there was a positive and statistically strong correlation between the induction of LB and the deformation by heavy metals (Pb and Se). Finally, based on this study, it was concluded that high levels of metal stress led to increase in LB and valve deformation in *Gomphonema pseudoaugur*. The deformation of diatoms, which can be used as an important indicator of biological monitoring and important parameter for the production of biofuels [89]. These deformed frustules can be effective indicators of water quality of wastewaters. Antoni *et al.* [104] found big LB in diatom cells treated with Zn compared to the control ones, and found that AgNP enhanced LB in *Thalassiosira* sp [105]. On the other hand [106], they observed no increase in LB in diatoms treated with Pb. [107]. They did a detailed study of teratological forms of diatoms under different environmental stresses making them a highly sensitive biosensor for all types of environmental pollutants [108]. This could be helpful in economical harvest of lipid/biofuel from diatoms also known as Diafuel^TM^ [109], unlike the difficulties faced by researchers in optimizing the economical yet viable way of harvesting biofuel from diatoms using centrifugation, pulse electric field and resonance energies [110]. Besides teratological changes in diatoms there was decrease in chlorophyll pigments on exposure to trace metals [106,111,112], although it increased in some cases [113] whereas no changes were observed in many other cases [114]. Not limiting to this there is an alteration in the physiology as well organ structure of diatom on exposure to heavy metals. Diatom motility, nucleus anomalies have been observed in diatoms exposed to heavy metals. It has been observed in *Fallacia pygmaea* and *Navicula novaesiberica* there was damage in the nuclear membrane as well as abnormal nucleus location on exposure to Cr (IV) for 7 days [115].

## 3. Diatom as SiO_2_-nanoshells in conjunction with ligands to remediate wastewater

It is quite evident that the silica shells of the diatoms protect them from adverse environmental stresses. These encapsulated nano shells can be functionalized or modified without changing their abiotic properties by incorporating or doping of some elements and metal alkoxides *via* numerous processes of silicification [116,117]. Functionalization of natural silica shell is effortlessly attained by coupling of the surfaces silanol with suitable ligands on to their surface which is rich in -OH, -NH_2_, -COOH, -Si and other functional groups [118,119]. Moreover, several studies support that this rigid silica encapsulates biomolecules such as enzymes, lipids, protein, and other cell components, providing thermal stability and protection of the diatom cells from any aggressors *viz*; surfactant agent, cell lysis enzymes, pathogens, *etc.* [120]. This encapsulation concept can easily facilitate the silica nanoparticles on the cell surface which is extensible to any subjective nanoparticle to form the ligand chemistries [121]. The diatoms, silica cell wall owing to its porous structure permits the immobilization of target molecules onto its frustule surface as well as inside the pores, thus allowing the doping of sensing molecules, with high sensitivity and immediate responses [109,122]. In recent studies, various biosensors with ultrahigh sensitivity using diatom bio-silica, peptides and nanoparticles have been used in proteins self-assembly on Ag and Au NP on pores in diatom surface which under the laser beam undergo heating and thus release of bioenergy [123,124]. Its uniform porous architecture not only give it a porous sponge with wide surface area but is also photoluminescent which is an analog of crystalline silicon. Researchers have developed photoluminescence-based diatom biosensors for sensing the nitroaromatic compounds like 2, 4, 6-trinitrotoluene (TNT) [125]. Thus, adsorbents with essential parameters such as quick adsorption, easy separation, high adsorption capacity, equilibrium time, and with high stability are generally used for treatment of wastewater [126].

### 3.1 Heavy metals

Since diatoms synthesize antioxidant compounds such as polyunsaturated fatty acids (PUFA), terpenes, pigments and carotenoids like fucoxanthin to control uptake, detoxification, transport, and accumulation of heavy metals [37,127]. Photosynthetic pigments like chlorophyll a, b, and c are influenced by heavy metal toxicity, but pigment from diatoms enhance the detoxification of heavy metals. According to the study by Corcoll et al [128], a fast bioaccumulation of Zn after few hours of exposure in Osor stream, Spain produced different damaging effects on the biofilm assessed *via* diatom indices. This was associated with reduced photosynthetic efficiency effecting the photosynthetic pigments which depends on cell surface adsorption, intracellular, and extracellular accumulation of the heavy metals. Cell death generally takes place when the metals reach intracellular cavities of cell. After longer time of exposure there were changes in Zn and Fe bioaccumulation and concentration of Zn in water sites which were related to diatom community changes; decline in diatom cell biovolume and chlorophyll pigment studies [128]. Another common hazardous heavy metal is Pb^2+^, which is commonly found in our environment and results in serious health problems like bone, kidney damages, brain, and liver disorders. Therefore, it is very necessary to reduce the levels of these toxic contaminants from our wastewaters before releasing into the environment [129,130]. To overcome such issues a synthetic approach is usually taken to prepare conjugate hybrid complex structures by combining metal organic frameworks (MOFs) and diatoms. By using Metal-organic framework [MIL-100(Fe)] new hybrid MIL100(Fe)-diatomaceous earth a MOF was prepared for the very first time having a high surface area to remove Pb^2+^. Thus, the MOF of MIL-100(Fe)-diatomaceous earth showed removal efficiency of Pb^2+^ by 96.45% at adsorption rate of 120 min while simultaneously possessing high adsorption capacity of 155 mg/g [131]. So it is noteworthy that diatoms are capable for adsorbing heavy metal ions and trace metals from wastewater [132]. They have the capability to adsorb and desorb metals by metal binding functional groups on the siliceous cell surface forming the intracellular ligands with trace metals [133]. One such laboratory marine diatom which is potential candidate for laboratory experiments is *Phaeodactylum tricornutum* which shows the ability to adsorb the Cd metal ions and form the metal ligand [134]. Hernández-Á vila in 2017 reported absorption of Pb, Cd, and Cu by antioxidant enzymes and phytochelations [135]. *Nitzschia* was reported to adsorb the Zn, Cu, Co, and Mn chiefly by hydrogen bonding and van der Walls interactions. It demonstrates the adsorption of Cd by linking the shell surface [136–138]. *Planothidium lanceolatum* forms the ligands with metals like Cd, Cu, and Zn by adsorption and absorption of metals *via* π−π interactions [137]. Absorption of Cd in *Thalassiosira weissflogii* and *Thalasssiosira oceanica* by metallothione in transport system *via* chelation effect mechanisms and physical interactions and chemical bonding, respectively, was observed. While *Thalassiosira pseudonana* demonstrates the chemical adsorption of Zn, Cd, and Cu metal ions [134,139], reported that *Chaetoceros costatum* has high affinity for hydrogen bonds thus absorb heavy metals like Hg [140].

### 3.2 Dyes

**Figure 2.**
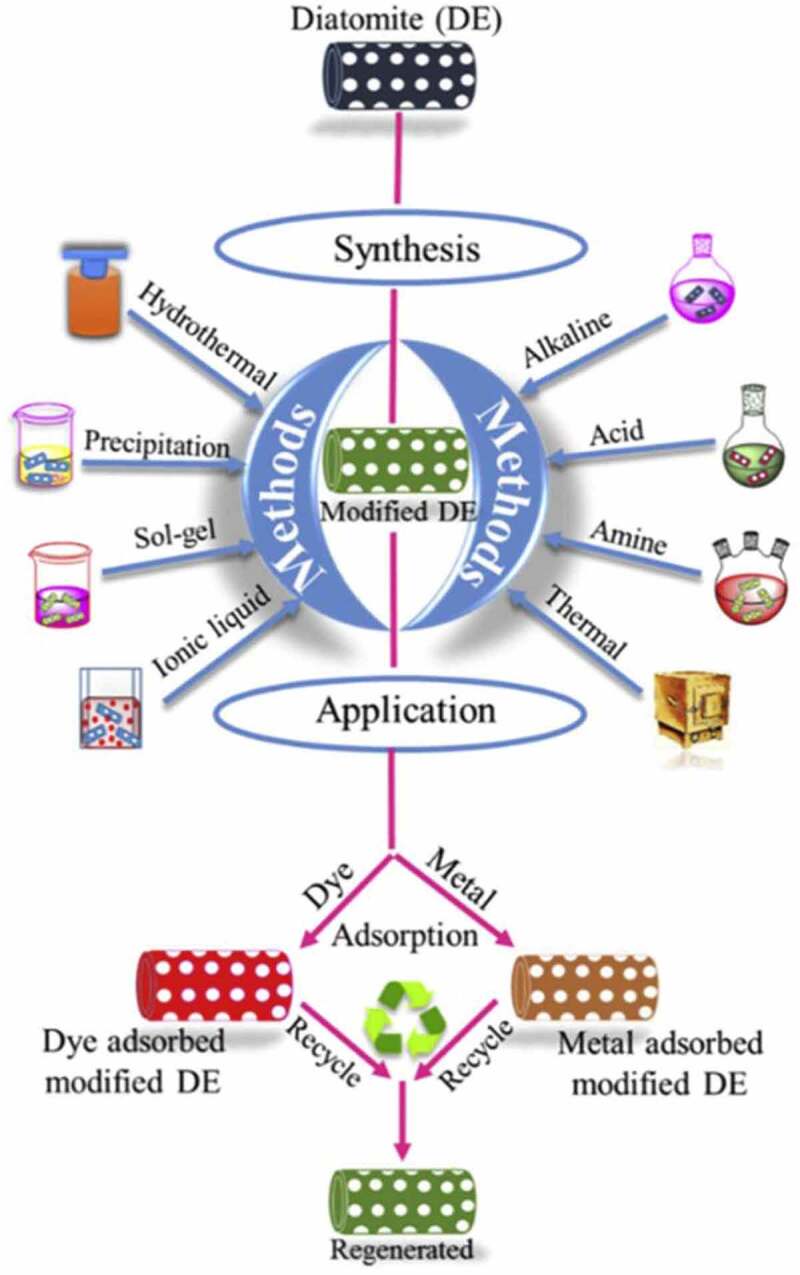
Schematic representation of surface modification and treatment of DE, and their application in the removal of dyes and metal ions along with regeneration. Reproduced with permission from [145]

Dyes are toxic, mutagenic, carcinogenic in nature and are also non-biodegradable due to their aromatic structure. Synthetic dyes are widely used in the leather, cosmetic, paper, plastics, and textile industries [141,142]. Bio removal of these dyes such as azo dyes, acid dyes, cationic dyes, and others has gained more consideration due to their highly tenacious and xenobiotic properties [143]. When these chemicals are disposed in water stream leading to an upsurge in water pollution and change in aquatic life. The research showed that SiO_2_ in diatom frustule, which is a naturally available low cost bio-silica material, could be used for eriochrome black T (EBT) dye removal. To increase its efficiency, its surface is modified or engineered with epichlorohydrin followed by NH_4_OH so as to introduce amine functionality on the silanol groups on diatom surface. This could help in adsorbing the EBT dye selectively even in the presence of other interfering dyes. Henceforth, this was found to be an efficient adsorbent for the removal of EBT dye from the effluents of dye-based and textile industries [65]. Figure 2 shows surface modification of diatom and their role in removal of dyes and metal ions.

In yet another study, diatoms frustules or diatomaceous earth were modified into bio-inspired polydopamine (PDA), DE-PDA and decorated with silver nanoparticles (AgNPs) in-situ to be used as nanocatalyst for the catalytic application. This designed nanocatalyst has been used for the degradation of cationic methylene blue (MB) and anionic Congo red (CR) dyes showing their fast degradation [144].

Similarly nano structure of MnO_2_-coated diatomite (MOCD) was used to remediate the methylene blue and methyl orange dyes, which showed biocatalytic activity and fast degradation within 30 minute or less [144]. Thus, they have potential applications in the field of wastewater treatment, and pollution degradation. Furthermore, removal of reactive blue 160 dye was demonstrated by Sriram et al [145], using diatomaceous earth at pH 2 with high adsorption capacity of up to 7.96 mg g^-1^ for treatment of wastewater from textile industries.

Thus, there is high requirement of cost-effective techniques, sustainable using hybrid diatom materials for the removal of toxic heavy metal ions and dyes.

### 3.3 Hydrocarbons

Polycyclic Aromatic Hydrocarbons (PAHs) are environmental contaminants and is a vast global concern [146,147]. PAHs have a wide range of hydrocarbons with over 100 compounds identified, each containing at least two aromatic rings in their assembly [48,148]. Owing to its hydrophobic nature, PAHs tend to accumulate in the aquatic sediments, results in its bioaccumulation and increased concentrations over time [149–151].

Diatoms are described to uptake polychlorinated biphenyls congener 2, 2′, 6, 6′-tetrachlorobiphenyl under standard nutrient availability conditions [143,152]. Due to the severe toxicity by polyaromatic hydrocarbons (PAHs), bioremediation has very limited success rate; however, marine diatoms *Skeletonema costratum* and *Nitzschia sp*. have revealed the quick biodegradation of hydrocarbons like phenanthrone (PHE) and fluoranthene (PLA), found commonly from marine sediments [153]. Furthermore, diatoms generate oxygen that can be utilized for the degradation of poly aromatic hydrocarbons, phenolic groups, and organic groups from the environments.

Research has shown the effects of three PAHs, *viz;* fluoranthene, pyrene, and benzo pyrene, on diatom *Thalassiosira pseudonana*, another common laboratory candidate diatom that is generally found globally in ocean and freshwaters. Modification at gene expression level by real-time PCR can be further investigated as the result of four chosen concentrations obtained from growth inhibition curves. According to the results, two out of eight genes were strongly influenced by the PAHs treatments. These were *lacsA* (involving the fatty acid metabolism) and *sil3* (involving the formation of the silica shell). The observations showed that, it can be assumed that PAHs impaired the fatty acid metabolism and silica shell formation [154].

155 Li et al grew diatom *Stephanodiscus hantzschii* in titanium (IV) bis (ammonium lactato) dihydroxide solution in a continuous flow system. This integrated 7.2 ± 1.4% (mass fraction) of titanium in the diatom cell wall and thus forming silica-titania frustules and henceforth reported the photodegradation of three different types of pharmaceuticals and personal care products *viz*; triclosan, BPW and N, N-Diethylmeta-toluamide. The silica-titania frustules with hierarchical nano/microstructures which served as a prefiltration unit by selectively allowing pharmaceuticals and personal care products to pass through their nanopores and are therefore fit for the applications in nanotechnology and in environmental remediation [155].

## 4. Diatoms in microfluidics to treat wastewater

**Figure 3.**
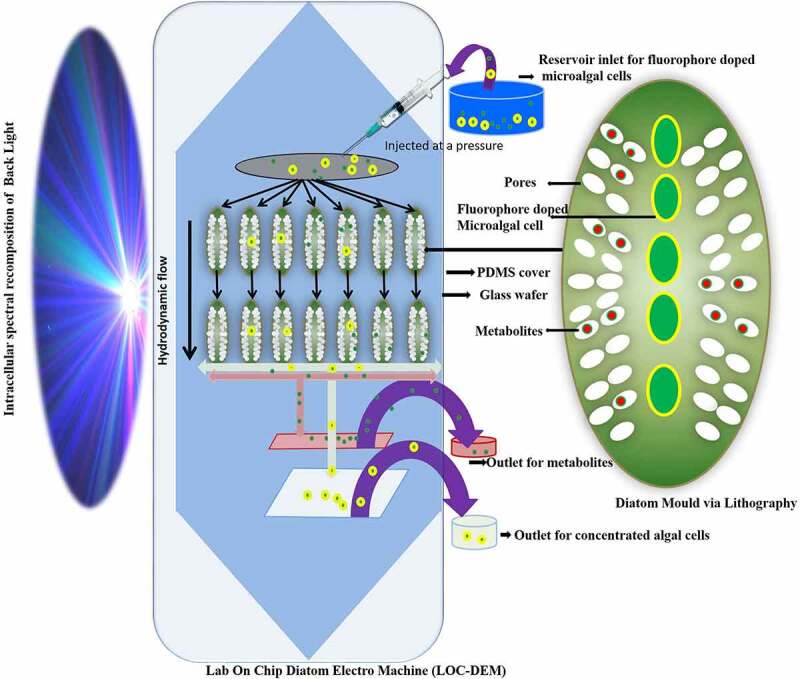
Lab on Chip Diatom Electro Machine (LOC-DEM) to remove wastewater pollutants while clean separating the algal concentrate flowing at hydrodynamic flow under the influence of intracellular spectral recomposition of light

Among microalgae, diatoms due to nanoporous silica wall are suitable material to be used in fabricating micro and nanosized devices. They acting as a photobioreactor for production of value added products mainly biofuel and carotenoids and simultaneously adsorbing the toxic metals, dyes and other pollutants present in the wastewater added as nutrient supply for their growth [156]. In one such study, it was found that a microfluidic diatomite analytical device (μDADs) was fabricated to remove illicit drugs [122]. This has been inspired from microfluidic paper devices which are capable to remove heavy metals and other analytes like glucose effectively and economically [157]. This led to fabrication of μDADs which were constructed on a simple glass slide with diatomaceous earth spin coated in a mixture of 0.4% of carboxymethyl cellulose at an area of 400 × 30  μm^2^. It has been used as lab on chip chromatography in conjunction with surface enhanced Raman spectroscopy (SERS) sensing protocol to separate cocaine (C_17_H_21_NO_4_). The lab on chip (LOC) device can not only detect toxic drugs but also mixture of drugs in any aqueous solution and even wastewater *via* capillary forces operating in μDADs. Besides this other toxic compound like PAH for eg. pyrene, 4-mercaptobenzoic acid (MBA) are nuisance in our environment which can also be removed by μDADs, which is a novel way for economic route to use microfluidics in the field of nanotechnology and nanosciences for remediation of toxic compounds from wastewater environments. Furthermore, fabricating a lab on chip diatom electromachine (LOC-DEM) model on a glass wafer may direct a hydrodynamic flow of fluorophore doped algal cells kept under pressure in an intracellular spectral recomposition of light as seen in Figure 3. The lipophilic fluorophore further enhances the photosynthesis and thus rate of synthesis of petrochemical and bioactive components. The separation of these metabolites and bacterial contaminants further takes place *via* pores of size as small as 5–10 nm. This will not only separate the algal concentrate which can be re-fed in fresh media for manufacture of more bioactive and petrochemical products but the different fluorophores sensitive to reactive oxygen species serve as pollutant indicators too. This type of microfluidic system helps in overcoming dewatering the microalgae a crucial step during algal biomass processing, sensing the pollutants like herbicides, phenols, metals, and biopolymers and harvesting enhanced valuable biometabolites under the influence of intracellular spectral recompositing of light.

## 5. Drawbacks and future perspectives

Drawbacks of usual methods for wastewater treatment make it difficult to develop a continuous treatment for wastewater disposal and recycling. So, it is very essential to find an alternative that would be high on resource recycling which take less energy and encourage economical circular bio-refinery concepts. Thus, microalgae can be a good surrogate for wastewater treatment [158].

Even though, microalgae utilize the nutrients from wastewater and not only recycle the wastewater but also help in remediating the toxic pollutants from it. This makes the wastewater reusable for agricultural and washing purpose. This is deficiently economical compared to the expensive water treatment methods in which industries dump their waste into oceans, rivers and lakes deteriorating the healthy flora and fauna. On the other hand, the use of microalgae has significant applications if they are used in economical microbial fuel cells (MFC) to not only clean the wastewater [159], but also produce electricity besides producing value added products like biofuel, carotenoids, proteins, carbohydrates, and polyhydroxyalkanoates [160,161]. Furthermore, diatoms along with other existing microalgae are responsible for synthesizing nanoparticles and synthesize various value added products while remediating wastewater employing various techniques including microbial fuel cells [162,163]. They thus behave differentially to different pollutants as seen in a case where diatom *Nitzschia palea* was exposed to seven different pollutants *viz*; paint, β propranolol drugs, sewage water, metasilicates, *Eichhornia crassipes*, detergents, hydrocarbons like petrol. It was found that diatom grew best in sewage water at a dilution of 5 mg and 10 mg/L, silica (60–120 µl/L), and petrol (5–15 µl/L) with moderate increase in lipid percentage [164]. Hydrocarbons at higher concentration than this are detrimental to microorganism growth and have different set of bacterium in it to valorize the soil petroleum sludge [27].

Although many different diatom based fuel cells besides MFC like diatom dye sensitized solar cells have been made to generate electricity by metabolically engineering diatoms with TiO_2_ nanoparticles using electrolyte and photosensitive dye [123,165]. Such fuel cells besides yielding biofuel need to be up scaled utilizing wastewater instead of electrolyte or even cost-effective nutrient media. Hence it is still challenging to researchers to find an economic and effective wastewater treatment methods that can be utilized and accepted globally [161].

## 6. Conclusions

Diatoms can be considered as sea jewels due to their rigid silica cell wall, which resists decomposition and are rich in value-added products. They besides cleaning, decrease 25% of our atmospheric CO_2_, thus reducing global warming. The diatoms frustule is made up of porous structure that has large surface area due to uniform nanoarray pattern and thus serves as nanoadsorbent for toxic metals and dyes. Even though live diatom cells equally adsorb the toxic pollutants but the live cells show deformities and abnormalities in their morphology and many times the cell undergo lipid stress thus resulting into high lipid bioaccumulation. Furthermore the nanoarchitecture of dead diatoms makes them an excellent choice to be used in microfluidic devices to sieve drugs and metals of varied sizes and shapes. Thus, biosensing the pollutants by diatoms living or dead at nanoscale level makes them a smart nanocontainers to biosense the wastewater rich metal and other pollutants.
